# Development of the Reading Comprehension Strategies Questionnaire (RCSQ) for late elementary school students

**DOI:** 10.3389/fpsyg.2022.1016761

**Published:** 2023-01-04

**Authors:** Rielke Bogaert, Emmelien Merchie, Yves Rosseel, Hilde Van Keer

**Affiliations:** ^1^Department of Educational Studies, Ghent University, Ghent, Belgium; ^2^Department of Data-analysis, Ghent University, Ghent, Belgium

**Keywords:** reading comprehension, reading strategy use, late elementary education, factor analyses, questionnaire development

## Abstract

Late elementary education constitutes a critical period in the development of reading comprehension strategies, a key competence in today’s society. However, to date, appropriate measurements to map late elementary students’ reading strategies are lacking. In this respect, the present article first describes the development and validation of the 26-item reading comprehension strategies questionnaire (RCSQ). To this aim, exploratory (sample 1: *n* = 1585 students) and confirmatory (sample 2: *n* = 1585 students) factor analyses were conducted. These analyses resulted in the RCSQ, consisting of five subscales: (1) overt cognitive reading strategies, (2) covert cognitive reading strategies, (3) monitoring, and (4) evaluating. For non-native and bilingual students, a fifth subscale ‘using home language in view of comprehending texts’ emerged. Second, multilevel analyses were performed to explore individual differences in late elementary students’ reading comprehension strategy use. Implications for practice and future research are discussed.

## 1. Introduction

Reading comprehension is a key competence for students’ success in school (e.g., academic achievement) as well as in life (e.g., job success). Moreover, poor reading comprehension is negatively correlated with students’ learning performance, problem solving skills, and their future school and work career (OECD, 2018; Nanda and Azmy, 2020). Reading comprehension is defined as a complex and multifaceted process of creating meaning from texts (van Dijk and Kintsch, 1983; Castles et al., 2018). Notwithstanding the acknowledged importance of reading comprehension, many children struggle with it in late elementary education (i.e., fifth-and sixth-graders), especially when it comes to understand expository texts (Rasinski, 2017). According to both national and international studies, Flemish students are not performing well in reading comprehension (Tielemans et al., 2017; Support Center for Test Development and Polls [Steunpunt Toetsontwikkeling en Peilingen], 2018). More particularly, the results of a study on a representative sample of Flemish sixth-graders showed that 16% of the students failed to achieve basic reading comprehension standards (Support Center for Test Development and Polls [Steunpunt Toetsontwikkeling en Peilingen], 2018). Further, the study of Dockx et al. (2019) indicated that the fourth-grade results of high performing countries are only achieved by sixth-grade Flemish students. This is alarming, since poor reading comprehension can negatively influence access to higher education and fruitful education (Wigfield et al., 2016). Moreover, late elementary education is a critical period to develop appropriate expository text comprehension skills (Keresteš et al., 2019). Reading comprehension strategies play an essential role in effective comprehension of expository texts (Follmer and Sperling, 2018). Unfortunately, to date only few studies addressed the challenge of mapping students’ reading strategy repertoire precisely in this critical period. This might be due to the lack of reliable and valid measurement instruments attuned to this age group. Therefore, the present study aimed to fill this gap by developing an age-appropriate expository reading comprehension strategies questionnaire. Additionally, individual differences in Flemish late elementary students’ actual strategy use (i.e., according to their gender, grade, achievement level, and home language) are examined by means of this questionnaire.

## 2. Reading comprehension strategy use

### 2.1. Theoretical and empirical background

Reading comprehension strategies have already been defined in many ways. However, in general, the following aspects are unanimously stressed: (1) the active role of proficient readers (Garner, 1987; Winograd and Hare, 1988), (2) the relevance of deliberate and planful activities during reading (Garner, 1987; Afflerbach et al., 2008), and (3) the aim to improve text comprehension (Graesser, 2007; Afflerbach et al., 2008). Essentially, reading strategies are consequently described as deliberate and planful procedures with the goal to make sense of what is read, in which readers themselves play an active role.

Prior research conceptually refers to several reading strategy classifications, i.e., according to strategies’ perceptibility, their approach, their goal, or their nature. Kaufman et al. (1985) classification is based on the perceptibility, differentiating between overt, observable (e.g., taking notes) and covert, non-observable strategies (e.g., imagining). However, this classification rarely occurred in subsequent research. A recent meta-analysis of Lin (2019) refers to research relying on the three other classifications. First, based upon the approach, Block (1986) and Lee-Thompson (2008) distinguish bottom-up (e.g., decoding) and top-down strategies (e.g., using prior knowledge). Second, Mokhtari and Sheorey (2002) classify reading strategies regarding their goal; distinguishing global (e.g., text previewing), problem-solving (e.g., rereading), and support (e.g., highlighting) strategies. Third, as to their nature, cognitive (e.g., summarizing) and metacognitive (e.g., setting goals) strategies have been distinguished (Phakiti, 2008; Zhang, 2018b). Additionally, motivational strategies (e.g., the use of positive or negative self-talk) are referred to as a third component of this last classification (e.g., O’Malley and Chamot, 1990; Pintrich, 2004).

Empirical studies on reading strategy use have already focused on different target groups (e.g., children with autism; Jackson and Hanline, 2019; English Foreign Language learners; Habók and Magyar, 2019), in various subject domains (e.g., English language course; Shih et al., 2018; history; ter Beek et al., 2019), and at different educational levels (e.g., university level; Phakiti, 2008; secondary school students; Habók and Magyar, 2019). These studies mainly focus on reading strategy instruction (e.g., ter Beek et al., 2019) or on the relationship between reading strategy use and comprehension (e.g., Muijselaar et al., 2017). In this respect, good comprehenders appear to handle the use of strategies more strategically and effectively (Wang, 2016; Zhang, 2018b). Furthermore, good comprehenders dispose of a wide repertoire of strategies and are able to use these adaptively depending on the situation (Cromley and Wills, 2016). On the other hand, the research of Seipel et al. (2017) concludes that both poor and good comprehenders engage in a range of comprehension strategies, adapted moment-by-moment. The difference between both groups concerns which strategies are used (e.g., good comprehenders engage in more high-level processes such as monitoring their progress; Lin, 2019), but also when these are used (e.g., good comprehenders make more elaborations toward the text’s end). Finally, several studies examined students’ reading strategy use in relation to student characteristics such as grade, gender, achievement level, or home language (e.g., Denton et al., 2015; Van Ammel et al., 2021). For example, the study of Denton et al. (2015) on secondary students reported higher strategy use for girls, older students, and high comprehenders.

However, research on late elementary students reading strategy use, and the relation between these students’ strategy use and student characteristics is missing, probably due to the lack of appropriate measurement instruments. In addition, the context of our increasingly diverse society (e.g., between 2009 and 2019 the number of students with a home language other than the instructional language increased from 11.7 to 19.1% in Flanders; Department of Education and Training, 2020) is not yet reflected in the currently available instruments. More specifically, existing instruments are designed to be used with either native (e.g., Mokthari and Reichard, 2002) or non-native speakers (e.g., Zhang, 2018b) instead of aiming at measuring students’ strategy use across diverse groups. Therefore, the present study focuses on the strategy use of native (i.e., students who have the instructional language as home language), non-native (i.e., students whose home language is not the instructional language), and bilingual students (i.e., students who use both the instructional language as another language at home).

### 2.2. Measuring reading comprehension strategies

The variety of definitions and classifications of reading comprehension strategies as found in the literature, is also reflected in the available instruments used to map these strategies. In this respect, eight current instruments are considered in our study (Carrell, 1989; Mokthari and Reichard, 2002; Dreyer and Nel, 2003; Phakiti, 2003, 2008; Zhang and Seepho, 2013; Shih et al., 2018; Zhang, 2018b). The classification according to the approach of reading strategies is reflected in the questionnaire of Carrell (1989) and Shih et al. (2018), while the Metacognitive Awareness of Reading Strategies Inventory (Mokthari and Reichard, 2002), which is currently the most used instrument, reflects the classification based upon the goal of reading strategies. Various strategy questionnaires (i.e., Phakiti, 2003, 2008; Zhang and Seepho, 2013; Zhang, 2018b) are based upon the classification regarding their nature. The classification according to the strategy’s perceptibility is not used in published reading strategy instruments; while on the other hand some researchers appear not to rely on any classification (e.g., Dreyer and Nel, 2003).

When overviewing the available instruments assessing reading comprehension strategy use, three important aspects should be noticed. First, different classifications are often used separately and are not integrated into a comprehensive framework or measurement instrument. Second, the previously published instruments mostly focus on older students (secondary education; e.g., Mokthari and Reichard, 2002; Shih et al., 2018; or higher education; e.g., Phakiti, 2008; Zhang, 2018b) and/or focused exclusively on the strategy use of non-native students (e.g., Phakiti, 2003, 2008; Zhang and Seepho, 2013; Shih et al., 2018). In sum, an instrument attuned to late elementary school students, also taking into account diversity in home language, is currently lacking. A third consideration concerns the measurement method. All abovementioned research uses self-reports to map strategy use. Self-reports are advantageous as the reading process is less disturbed then when using online measurement methods and they are relatively easy to implement on large-scale (Schellings and van Hout-Wolters, 2011; Vandevelde et al., 2013). Considering these advantages, there is a permanent need for reliable and valid self-report questionnaires (Vandevelde et al., 2013). On the other hand, it is questionable whether accurate answers can be collected from students with this method (Cromley and Azevedo, 2006), since they might rather provide insight in students’ perceptions than in their actual strategy use (Bråten and Samuelstuen, 2007). However, this concern might partly be met by using task-specific instruments (Schellings et al., 2013). The items of a task-specific instrument examine students’ particular skills or approach in the context of a just completed task (e.g., reading comprehension test), while a general instrument measures students’ skills or approach in general, independent of a particular task. In this respect, general instruments are considered in the literature as a poor predictor of actual strategy use, given the context-, domain-, and goal-dependence of reading comprehension strategies (Bråten and Samuelstuen, 2007). Therefore, task-specific instruments are believed to provide a better estimate of one’s own strategy use (Schellings et al., 2013; Merchie et al., 2014). Unfortunately, except for the instrument of Phakiti (2003, 2008), currently available questionnaires do not formulate items in a task-specific way (e.g., Shih et al., 2018; Zhang, 2018b).

In conclusion, a new task-specific self-report instrument for late elementary school students, including native, non-native, and bilingual speakers, is urgently needed to map their reading strategy use.

## 3. Objectives of the study

The first research objective (RO1) is to develop and validate a task-specific self-report questionnaire measuring late elementary school students’ expository reading comprehension strategies. The second research objective (RO2) is to examine individual differences in students’ strategy use (i.e., according to students’ gender, grade, achievement level, and home language) by means of this newly developed instrument.

## 4. Materials and methods

### 4.1. Participants

A total of 3,170 students (51.5% fifth-graders, 48.5% sixth-graders, 49.5% boys, 50.5% girls) from 163 classes in 68 different schools in Flanders (Belgium) participated. The average number of participating students was 46.62 (*SD* = 24.75) within schools and 19.45 (*SD* = 4.77) within classes. A convenience sample method was used to recruit the participants (i.e., inviting Flemish schools by e-mail to participate; response rate of 20.67%). The overall mean age of the students was 11.38 years (*SD* = 0.93). 87.3% of the students were native speakers of the instructional language (i.e., Dutch), 7.1% were non-native speakers (i.e., another language than Dutch as home language), and 5.6% were bilingual speakers (i.e., Dutch combined with another language as home language). The research was in line with the country’s privacy legislation as informed consent was obtained for all participants. In this respect, agreeing to participate via the informed consent was the inclusion criteria for the participants to be involved in the study.

### 4.2. Measurement instrument: Development of the reading comprehension strategies questionnaire

Due to the caveats detected in prior research (see section “Introduction”), a new task-specific measurement instrument was developed and validated (*cf*. RO1), consistent with the Standards for Educational and Psychological testing (American Educational Research Association, American Psychological Association, and National Council on Measurement in Education, 2014) and following prior research (e.g., Vandevelde et al., 2013; Merchie et al., 2014). Based on the guidelines regarding effective test development (Downing, 2006), a multistep process was applied. First, an item pool of 61 items was deducted from eight previously published measurement instruments on reading strategy use (Carrell, 1989; Mokthari and Reichard, 2002; Dreyer and Nel, 2003; Phakiti, 2003, 2008; Zhang and Seepho, 2013; Shih et al., 2018; Zhang, 2018b). Relevant items reflecting the different classifications (i.e., based upon the perceptibility, approach, goal, or nature; see introduction section) were selected to guarantee a diversity of strategies. More specifically, overt and covert (i.e., based upon the perceptibility), bottom-up and top-down (i.e., based upon the approach), global, problem-solving, and support (i.e., based upon the goal), and cognitive and metacognitive (i.e., based upon the nature) reading strategies were included. Further, the items were adjusted to the target group by simplifying words, phrases, or sentences (e.g., “to increase my understanding” was changed into “to better understand the text”). Second, to ensure content validity, the items were reviewed for content quality and clarity by two experts on reading comprehension and instrument development, and by a primary school teacher, resulting only in minor word modifications. For each item the experts debated whether the item was essential, useful, or not necessary (*cf*. Lawshe, 1975) to ensure reading comprehension strategies are covered in their entirety. In consultation with the teacher the word usage of the questionnaire was aligned with the classroom vocabulary (e.g., “section” was changed into “paragraph”). Third, one fifth grade class (*n* = 22) identified confusing words during answering the RCSQ items to avoid construct-irrelevant variance (i.e., examine the comprehensibility and possible ambiguity of the items). After this step, the instrument consisted of 58 items for all students and 3 additional items for non-native and bilingual students (61 items in total).

Items were alphabetically sorted under the titles “before reading,” “during reading,” and “after reading” in order to present the RCSQ items in a logical sequence to the students, as advised by the Flemish Education Council (Merchie et al., 2019). However, the subscales emerging from the exploratory factor analyses (EFA) and confirmatory factor analyses (CFA) analyses were used to calculate students’ scores on the RSCQ and not the abovementioned threefold division in the presentation for the students. Each item was scored on a 5-point Likert scale, ranging from 1 (completely disagree) to 5 (completely agree). As substantiated above, developing a task-specific RCSQ was opted for. Therefore, the items referred to a reading comprehension task administered beforehand. This task was representative for a reading comprehension lesson in Flemish education (i.e., reading three short expository texts, followed by 8–12 content-related questions). The items of the RCSQ are expressed in the past tense and explicitly make a link between the questioned strategies and the reading comprehension task (e.g., “Before I started reading, I first looked at the questions”).

### 4.3. Procedure

The reading comprehension task and RCSQ were administered in a whole-class setting in Dutch during a 50-min class period in the presence of a researcher and the teacher. The students also received a background questionnaire to map their individual student characteristics (e.g., gender, home language). Students’ achievement level was mapped by means of a teacher rating, since experienced teachers can make accurate judgments to this respect (Südkamp et al., 2012). More specifically, teachers indicated their students as high, average, or low achievers.

### 4.4. Data analysis methods

#### 4.4.1. RO1: Parallel, exploratory, and confirmatory factor analyses, measurement invariance tests, and reliability analyses

In view of RO1, parallel analyses (PA), exploratory factor analyses (EFA), confirmatory factor analyses (CFA) and reliability analyses were conducted to develop and validate the reading comprehension strategies questionnaire.

##### 4.4.1.1. Parallel and exploratory factor analyses

First, the sample was split using SPSS 25 random sampling, to execute EFA (*n* = 1585) and CFA (*n* = 1585) in two independent subsamples. Chi-square analyses shows no significant differences between both samples in terms of students’ gender (χ2 = 0.016, df = 1, *p* = 0.901), grade (χ2 = 0.182, df = 1, *p* = 0.670), achievement level (χ2 = 2.171, df = 2, *p* = 0.338), and home language (χ2 = 2.673, df = 2, *p* = 0.263).

Second, PA and EFA were iteratively conducted on the first sample (*n* = 1585) in R 3.6.1 (R Core Team, 2014). PA was performed in order to determine the numbers of factors to be retained, using the 95th percentile of the distribution of random data eigenvalues and 1,000 random data sets (Hayton et al., 2004). The EFA was performed with the lavaan package 0.6–5 (Rosseel, 2012) using a robust Maximum Likelihood Estimator (MLR) for the non-normality of the data and geomin rotation to uncover the questionnaire’s underlying structure. Geomin rotation was opted for, since it can offer satisfactory solutions when a factor loading structure is too complex to analyze with other rotation methods (Hattori et al., 2017), as was the case in the present sample. Cluster robust standard errors were applied to account for the data’s nested structure. The number of factors was specified, based on the parallel analyses. Resulting models were iteratively compared, consisting of five-to eight-factor solutions. The following criteria were used to determine the best model fit: (a) significance level to select stable items (Cudeck and O’Dell, 1994), (b) deletion of items with factor loadings under .30 and cross loadings (Tabachnick and Fidell, 2013), and (c) theoretical relevance of the items.

##### 4.4.1.2. Confirmatory analyses

In a third step, CFA was executed on the second sample (*n* = 1585) in R, using the lavaan package, to examine whether the exploratory structure is consistent with the observed data (Schmitt, 2011). Moreover, CFA is a commonly used method to examine construct validity (e.g., Besnoy et al., 2016). Since no Flemish standardized reading comprehension test is available, criterion validity measuring the correlation between the RCSQ and reading comprehension was not examined in this study. The Yuan-Bentler (YB) χ2 statistic was used as a correction for the non-normality of the data, with a scaling correction factor of 1.138 (Yuan and Bentler, 2000). It is recommended to use multiple fit indices, since no single fit index can assess all aspects of goodness of fit at once (Sun, 2005). The following cluster robust fit indices were used: (a) comparative fit index (CFI), (b) Tucker−Lewis index (TLI), (c) root mean square residual (RMSR), and (d) root mean square error of approximation (RMSEA). CFI and TLI point to a reasonable to good fit by value above 0.90 or 0.95 (Little, 2013). Values less than 0.07 are acceptable for RMSEA (Steiger, 2007). Values lower than.08 are aspired for SRMR (Hu and Bentler, 1999).

##### 4.4.1.3. Measurement invariance tests

In a fourth step, measurement invariance tests were conducted to verify factor structure invariance across students’ gender, general achievement level, and home language (Cheung and Rensvold, 2002). More specifically, the baseline model was tested for the same factor structure across groups (i.e., configural invariance). The subsequent models tested more restrictions of the factor loadings: weak invariance (i.e., equal loadings) and strong invariance (i.e., equal loadings and intercepts).

##### 4.4.1.4. Reliability analyses

Finally, reliability analyses were conducted to explore the internal consistency of the subscales.

#### 4.4.2. RO2: Multilevel analyses

In light of RO2, multilevel analyses on the complete sample were conducted in MLwiN 3.02 given the data’s two-level hierarchical structure (level 1: students; level 2: classes). In this respect, the interdependency between students, belonging to the same class and sharing the same teacher, was taken into account (Maas and Hox, 2005). Subsequently, also student characteristics (i.e., gender, grade, achievement level, and home language) were included as independent variables in the model to explore individual differences in students’ strategy use (i.e., dependent variable). Standardized regression coefficients were calculated in order to better understand the relative impact of the significant parameters. More specifically, since standardized parameter estimates can be interpreted as effect sizes, Cohen’s benchmarks for interpretation of effect sizes were followed (i.e., small effect size: d = 0.2, medium effect size: d = 0.5, and large effect size: d = 0.8; Cohen, 1977).

## 5. Results

### 5.1. RO1: Development and validation of the reading comprehension strategies questionnaire

Parallel analyses (PA) and exploratory factor analyses (EFA) alternated iteratively. The first PA suggested eight factors. Subsequently, EFA was conducted on the complete set of 61 items. Five items were deleted based on the significance level to retain stable items. The newly obtained item set was re-analyzed through PA. This process was repeated five times, until all items loaded significantly on one of the factors. Thirty-one items over five factors remained. Several items that measured roughly similar strategies but were phrased slightly differently were omitted. For example, the item “I tried to understand the main idea in the text” was retained, while the item “I tried to understand the rationale of the text” was removed. In the next step, factor loadings were examined as well, and four items were deleted due to factor loadings under .30 and due to cross loadings. Additionally, one item was removed since the item did not load significantly and was of limited theoretical relevance. For this set of 26 items, PA suggested five factors. An acceptable factor structure appeared with the subsequent EFA on this item set: 24 items loaded significantly, had a factor level above 0.30, and were theoretically relevant. Notwithstanding the fact that two items did not load significantly, these items were retained given that these had a factor loading above .30 and given their theoretical relevance. For three items a small cross loading remained. However, based on prior research, these items were retained (e.g., Zahoor et al., 2017; Lohbeck and Petermann, 2019). According to Vichi (2017), cross loadings are justified as long as they ensure a significantly better model fit. In this respect, a five-factor model consisting out of 26 items was retained after five iterations (see Table 1). After the selection of these 26 items, the reading comprehension experts were reconsulted as a final step in the development process, to ensure content validity.

**Table 1:** Communalities, pattern and structure coefficients of EFA (sample 1): Significance level and factor loadings.

Item	OCOG			CCOG			MON			EVA			HL			
	Pattern	Structure	*p*-value	Pattern	Structure	*p*-value	Pattern	Structure	*p*-value	Pattern	Structure	*p*-value	Pattern	Structure	*p*-value	Communalities
OCOG1	**0.559**	**0.616**	**0.000**	0.296	0.491	0.074	0.065	0.267	0.462	0.007	0.026	0.898	0.049	0.206	0.597	0.449
OCOG2	**0.812**	**0.721**	**0.000**	0.176	0.413	0.426	0.012	0.206	0.838	−0.069	−0.075	0.408	0.055	0.224	0.628	0.538
OCOG3	**0.950**	**0.808**	**0.000**	−0.042	0.274	0.722	−0.105	0.076	0.366	−0.020	−0.098	0.680	−0.113	0.090	0.296	0.678
OCOG4	**0.894**	**0.759**	**0.000**	−0.259	0.184	0.031	0.148	0.249	0.351	0.095	−0.003	0.160	0.033	0.200	0.496	0.625
OCOG5	**0.867**	**0.789**	**0.000**	0.052	0.354	0.602	−0.145	0.076	0.296	0.081	0.010	0.204	0.009	0.195	0.881	0.643
OCOG6	**0.672**	**0.667**	**0.000**	0.235	0.399	0.301	−0.060	0.138	0.404	−0.184	−0.169	0.051	0.001	0.173	0.982	0.488
OCOG7	**0.596**	**0.615**	**0.000**	0.190	0.387	0.436	0.053	0.217	0.492	−0.115	−0.103	0.268	−0.055	0.114	0.450	0.411
CCOG1	0.059	0.172	0.550	**0.530**	**0.404**	**0.010**	0.047	0.180	0.721	0.032	0.106	0.710	−0.269	−0.124	0.067	0.204
CCOG2	0.018	0.243	0.845	**0.535**	**0.525**	**0.007**	0.132	0.297	0.402	0.028	0.125	0.732	0.027	0.114	0.835	0.288
CCOG3	−0.102	0.061	0.266	**0.384**	**0.453**	**0.012**	0.185	0.332	0.199	0.285	0.392	0.003	−0.088	−0.039	0.296	0.347
CCOG4	0.024	0.274	0.840	**0.658**	**0.567**	**0.000**	0.033	0.233	0.807	−0.110	0.016	0.406	0.006	0.108	0.949	0.332
CCOG5	0.061	0.277	0.650	**0.462**	**0.495**	**0.066**	0.269	0.377	0.092	0.066	0.140	0.562	0.154	0.211	0.220	0.307
CCOG6	0.035	0.203	0.708	**0.631**	**0.476**	**0.025**	−0.252	0.010	0.127	0.136	0.181	0.359	0.066	0.124	0.574	0.273
CCOG7	0.114	0.214	0.190	**0.305**	**0.401**	**0.151**	0.188	0.310	0.120	0.200	0.255	0.114	−0.169	−0.073	0.110	0.248
MON1	−0.024	0.168	0.691	0.061	0.321	0.744	**0.847**	**0.727**	**0.000**	0.062	0.142	0.512	0.054	0.110	0.536	0.536
MON2	0.157	0.243	0.220	−0.020	0.203	0.765	**0.724**	**0.548**	**0.000**	−0.218	−0.113	0.114	−0.035	0.052	0.606	0.343
MON3	−0.028	0.189	0.645	0.088	0.343	0.571	**0.896**	**0.765**	**0.000**	−0.025	0.079	0.620	0.058	0.120	0.534	0.592
EVA1	−0.071	−0.118	0.483	−0.014	0.003	0.916	−0.162	−0.114	0.177	**0.475**	**0.433**	**0.000**	0.101	0.053	0.294	0.225
EVA2	−0.075	−0.134	0.409	0.048	0.085	0.672	−0.040	0.011	0.604	**0.526**	**0.552**	**0.000**	−0.118	−0.147	0.134	0.329
EVA3	−0.061	0.018	0.536	0.206	0.304	0.175	0.050	0.175	0.485	**0.579**	**0.583**	**0.000**	0.099	0.102	0.334	0.389
EVA4	0.018	−0.076	0.778	−0.009	0.099	0.939	−0.088	−0.002	0.433	**0.809**	**0.731**	**0.000**	−0.074	−0.089	0.424	0.546
EVA5	0.058	0.102	0.378	0.206	0.337	0.137	0.116	0.240	0.376	**0.595**	**0.575**	**0.000**	−0.058	−0.012	0.390	0.397
EVA6	0.082	0.034	0.372	−0.042	0.132	0.735	−0.012	0.077	0.834	**0.715**	**0.640**	**0.000**	0.117	0.103	0.320	0.427
HL1	−0.015	0.217	0.635	0.441	0.326	0.209	−0.288	−0.038	0.136	−0.013	0.014	0.668	**0.841**	**0.622**	**0.000**	0.475
HL2	0.317	0.455	0.034	0.126	0.297	0.553	0.010	0.148	0.863	−0.112	−0.105	0.357	**0.679**	**0.652**	**0.000**	0.533
HL3	0.308	0.367	0.088	−0.054	0.192	0.367	0.066	0.147	0.441	0.078	0.022	0.548	**0.950**	**0.715**	**0.000**	0.557

OCOG, overt cognitive reading strategies; CCOG, covert cognitive reading strategies; MON, monitoring; EVA, evaluating; HL, using home language in view of comprehending texts. Selected items based on the EFA analysis are highlighted in bold.

Two CFA models were conducted on sample 2: (a) five-factor model based on the EFA analyses and (b) a higher-order model distinguishing cognitive and metacognitive strategies. The five-factor model revealed better cluster robust fit indices (YB χ2 = 891.547, df = 289, *p* < 0.001, CFI = 0.93, TLI = 0.92, RMSEA = 0.04, 90% CI [0.03, 0.04], SRMR = 0.06) than the cognitive-metacognitive model (YB χ2 = 985.228, df = 293, *p* < 0.001, CFI = 0.92, TLI = 0.91, RMSEA = 0.04, 90% CI [0.03, 0.04], SRMR = 0.07). No additional items had to be removed.

Based on small changes in the comparative fit index (i.e., ΔCFI smaller or equal than .01; Cheung and Rensvold, 2002) and overall satisfying goodness-of-fit indexes, measurement invariance tests indicate strong invariance for each student characteristic. This showed the stability of the RCSQ’s factor structure across students’ gender, general achievement level, and home language (Table 2; Vandenberg and Lance, 2000).

**Table 2:** Measurement invariance testing: Summary of the goodness-of-fit statistics.

Measurement invariance	Yuan-Bentler χ2	*df*	*p*-value	CFI	RMSEA		ΔCFI	Chi-square difference test *p*-value
**Gender**								
Configural invariance	1247.614	578	<0.001	0.921	0.040			
Weak invariance	1264.055	599	<0.001	0.922	0.40	Model 1 vs. model 2	0.001	0.571
Strong invariance	1320.185	620	<0.001	0.918	0.040	Model 2 vs. model 3	0.004	<0.001
**General achievement level**								
Configural invariance	1578.162	867	<0.001	0.914	0.042			
Weak invariance	1621.897	909	<0.001	0.914	0.041	Model 1 vs. model 2	0.000	0.313
Strong invariance	1732.054	951	<0.001	0.906	0.042	Model 2 vs. model 3	0.008	<0.001
**Home language**								
Configural invariance	1246.103	578	<0.001	0.922	0.040			
Weak invariance	1263.709	599	<0.001	0.922	0.040	Model 1 vs. model 2	0.000	0.478
Strong invariance	1292.342	620	<0.001	0.922	0.039	Model 2 vs. model 3	0.000	0.159

df, degrees of freedom; CFI, comparative fit index; RMSEA, root mean square error of approximation.

After consulting experts and based on the literature on reading strategies, the five factors or subscales were labeled as overt cognitive reading strategies, covert cognitive reading strategies, monitoring, evaluating, and using home language in view of comprehending texts (see Supplementary Appendix). More specifically, the items within each subscale were scrutinized regarding to their content to decide which terminology would be used for each of them. For example, the first subscale was labeled as overt cognitive reading strategies since all items of this subscale were cognitive strategies that are observable (e.g., highlighting, making a summary). This last subscale was only administered with non-native and bilingual students (*n* = 494; sample 1: *n* = 257; sample 2: *n* = 237). Factor correlations are provided in Table 3. Descriptive analyses and Bentler’s rho reliability coefficients are provided in Table 4.

**Table 3:** Factor correlation matrix of CFA.

	OCOG1	CCOG1	MON1	EVA1	HL1	OCOG2	CCOG2	MON2	EVA2	HL2
OCOG1	1									
CCOG1	0.424	1								
MON1	0.228	0.372	1							
EVA1	−0.073	0.191	0.115	1						
HL1	0.240	0.165	0.089	−0.023	1					
OCOG2						1				
CCOG2						0.509	1			
MON2						0.352	0.605	1		
EVA2						−0.011	0.487	0.247	1	
HL2						0.303	0.332	0.343	0.173	1

1, correlation matrix of CFA in sample 1; 2, correlation matrix of CFA in sample 2; OCOG, overt cognitive reading strategies; CCOG, covert cognitive reading strategies; MON, monitoring; EVA, evaluating; HL, using home language in view of comprehending texts.

**Table 4:** Descriptive statistics of the RCSQ subscales (sample 2).

	*M*	*SD*	n_items_	Bentler’s ρ
Overt cognitive reading strategies	1.68	0.71	7	0.84
Covert cognitive reading strategies	2.93	0.72	7	0.66
Monitoring	2.88	1.09	3	0.74
Evaluating	3.60	0.71	6	0.75
Using home language in view of comprehending texts	2.33	1.12	3	0.79

### 5.2. RO2: Examining individual differences in students’ reading comprehension strategy use

Because both student-and class-level variances of all the RCSQ subscales, with the exception of ‘using home language in view of comprehending texts,’ were significantly different from zero (see null models in Tables 5, 6), multilevel analyses were required. More specifically, for overt cognitive reading strategies, covert cognitive reading strategies, monitoring, and evaluating, respectively, 7.98%, 5.94%, 3.41%, and 2.12% of the variance is due to class-level differences, while 92.02%, 94.06%, 96.59%, and 97.88% of the variance is due to student-level differences. The constant in the null models in Table 5 represents the overall mean for all children in all classes, respectively for overt cognitive reading strategies (*M* = 1.69), covert cognitive reading strategies (*M* = 2.93), monitoring (*M* = 2.92), and evaluating (*M* = 3.60). The null model in Table 6 presents the reported mean for ‘using home language in view of comprehending texts’ (*M* = 2.37) for non-native and bilingual students.

**Table 5:** Summary of the model estimates.

Fixed part	Overt cognitive reading strategies		Covert cognitive reading strategies		Monitoring		Evaluating	
	Null model	Final model	Null model	Final model	Null model	Final model	Null model	Final model
CONS	1.69 (0.022)***	1.76 (0.035)***	2.93 (0.019)***	2.91 (0.035)***	2.92 (0.025)***	2.81 (0.049)***	3.60 (0.016)***	3.48 (0.030)***
Gender (girl)^a^	–	−0.003 (0.025)	–	0.036 (0.026)	–	0.282 (0.039)***	–	−0.016 (0.025)
Grade (sixth grade)^b^	–	−0.126 (0.038)**	–	0.008 (0.036)	–	−0.063 (0.049)	–	0.116 (0.029)***
Achievement level (low achievers)^c^	–	0.196 (0.037)***	–	0.034 (0.038)	–	0.095 (0.057)	–	−0.095 (0.036)**
Achievement level (high achievers) ^c^	–	−0.133 (0.029)***	–	−0.046 (0.030)	–	−0.065 (0.045)	–	0.201 (0.029)***
Home language (non-native students) ^d^	–	0.224 (0.053)***	–	0.087 (0.054)	–	0.034 (0.081)	–	−0.125 (0.051)*
Home language (bilingual students) ^d^	–	0.111 (0.048)*	–	0.098 (0.049)	–	0.079 (0.073)	–	−0.008 (0.047)
**Random part**	Null model	Final model	Null model	Final model	Null model	Final model	Null model	Final model
Level: class								
Class-level variance	0.054 (0.009)***	0.039 (0.007)***	0.032 (0.006)***	0.030 (0.006)***	0.037 (0.011)***	0.040 (0.011)***	0.016 (0.005)***	0.010 (0.004)**
Level: student								
Student-level variance	0.466 (0.012)***	0.450 (0.012)***	0.479 (0.013)***	0.475 (0.013)***	1.171 (0.031)***	1.134 (0.030)***	0.478 (0.013)***	0.461 (0.012)***
Loglikelihood	6471.290	6176.438	6458.115	6266.966	9326.786	8994.791	6529.320	6231.050
Number of units: class	163	163	163	163	163	163	163	163
Number of units: students	3031	2954	3011	2935	3088	3008	3072	2992

^a^Boys as reference category; ^b^Fifth grade as reference category; ^c^Average achievers as reference category; ^d^Native students as reference category. Standard error estimates are placed between brackets. ***p < 0.001, **p < 0.01, *p < 0.05.

**Table 6:** Summary of the model estimates regarding the RCSQ subscale ‘using home language in view of comprehending texts.’

Fixed part	Using home language in view of comprehending texts	
	Null model	Final model
CONS	2.37 (0.052)***	2.605 (0.130)***
Gender (girl)^a^	–	−0.037 (0.103)
Grade (sixth grade)^b^	–	−0.108 (0.112)
Achievement level (low achievers)^c^	–	0.232 (0.123)
Achievement level (high achievers)^c^	–	−0.339 (0.127)**
Home language (bilingual students)^d^	–	−0.227 (0.108)*
**Random part**	Null model	Final model
Level: class		
Class-level variance	0.019 (0.039)	0.051 (0.045)
Level: student		
Student-level variance	1.218 (0.086)***	1.114 (0.083)***
Loglikelihood	1506.679	1344.240
Number of units: class	135	130
Number of units: students	494	450

^a^Boys as reference category; ^b^Fifth grade as reference category; ^c^Average achievers as reference category; ^d^Non-native students as reference category. Standard error estimates are placed between brackets. ***p < 0.001, **p < 0.01, *p < 0.05.

In view of RO2, the role of individual differences was investigated (i.e., according to students’ gender, grade, achievement level, and home language). For each RCSQ subscale, the multilevel null model was further expanded (see final models in Tables 5, 6). The significant results are reported below as well as the effect sizes, ranging from 0.041 to 0.492 (i.e., small to medium effect sizes). As to gender, girls reported using significantly more monitoring strategies (*χ2* = 51.286, *df* = 1, *p* < 0.001, *SD* = 0.256) than boys. As to grade, fifth-graders report significantly more overt cognitive reading strategies (*χ2* = 10.799, *df* = 1, *p* = 0.001, *SD* = 0.175) and significantly fewer evaluating strategies (*χ2* = 15.792, *df* = 1, *p* < 0.001, *SD* = 0.166) than sixth-graders. As to achievement level, low achievers compared to average achievers, report significantly more overt cognitive reading strategies (*χ2* = 28.562, *df* = 1, *p* < 0.001, *SD* = 0.273) and significantly fewer evaluating strategies (*χ2* = 6.846, *df* = 1, *p* = 0.009, *SD* = 0.136). The reverse applies to high achievers compared to average and low achievers, reporting significantly fewer overt cognitive reading strategies (resp. *χ2* = 21.314, *df* = 1, *p* < 0.001, *SD* = 0.185; *χ2* = 86.957, *df* = 1, *p* < 0.001, *SD* = 0.458) and significantly more evaluating strategies (resp. *χ2* = 49.619, df = 1, *p* < 0.001, *SD* = 0.286; *χ2* = 71.772, *df* = 1, *p* < 0.001, *SD* = 0.422). Further, high achievers compared to low achievers, report significantly fewer covert cognitive reading strategies (*χ2* = 4.910, *df* = 1, *p* = 0.03, *SD* = 0.112). As to home language, both non-native and bilingual students reported using more overt cognitive reading strategies (resp. *χ2* = 18.171, *df* = 1, *p* < 0.001, *SD* = 0.311; *χ2* = 5.353, *df* = 1, *p* = 0.02, *SD* = 0.155) than native students. Further, native speakers report significantly more evaluating strategies (*χ2* = 5.991, *df* = 1, *p* = 0.01, *SD* = 0.178) than non-native students, who in their turn report significantly more monitoring (*χ2* = 8.425, *df* = 1, *p* = 0.004, *SD* = 0.041) than bilingual students.

Since only 494 students completed the items regarding ‘using home language in view of comprehending texts’ (i.e., non-native and bilingual students), the results for this subscale are presented separately (Table 6). High achievers and bilingual students reported using their home language in view of comprehending texts significantly less than average achievers (*χ2* = 7.138, *df* = 1, *p* = 0.008, *SD* = 0.295) and non-native students (*χ2* = 4.368, *df* = 1, *p* = 0.04, *SD* = 0.203). Additionally, the comparison of high and low achievers revealed that low achievers report a significantly higher use of this strategy (*χ2* = 18.152, *df* = 1, *p* < 0.001, *SD* = 0.492).

## 6. Discussion

The study’s first research objective was to develop a task-specific self-report questionnaire to map the reading comprehension strategy use of late elementary school students. Results of the EFA and CFA led to the RCSQ, containing five subscales (i.e., overt cognitive reading strategies, covert cognitive reading strategies, monitoring, evaluating, and using home language in view of comprehending texts). These subscales especially fit in with the classification based upon reading strategies’ perceptibility (i.e., overt versus covert; Kaufman et al., 1985) and nature (metacognitive versus cognitive; Lin, 2019). More particularly, the RCSQ subscales ‘monitoring’ and ‘evaluating’ can be theoretically regarded as metacognitive reading strategies, while the three remaining subscales are cognitive in nature. The other classifications referred to in the introduction section (i.e., based upon the approach and goal of reading strategies) are intertwined within the different subscales and not explicitly separated. For instance, the subscale covert cognitive reading strategies contains both bottom-up (e.g., “I read the first line of each paragraph to get the gist of the text”) and top-down strategies (e.g., “I tried to use what I already knew about the text topic to better understand the text”). In this respect, the RCSQ can be considered as a comprehensive questionnaire to measure students’ reading strategy use, covering strategies of various theoretical frameworks. However, as to metacognitive strategies, the literature frequently distinguishes between planning, monitoring, and evaluating strategies (e.g., Pintrich, 2004). However, in the present analyses, planning did not appear as a separate subscale, even though items reflecting planning were explicitly included in the original item pool (e.g., “I preview the text first by noticing its structure”). It might be that students immediately started reading the texts without any form of planning. For example, in the study of Botsas and Padeliadu (2003) fifth-and sixth-graders’ planning was low and even totally absent in students with reading disabilities. Therefore, it would be valuable in future research to explore whether or not and how students exactly make use of planning strategies during comprehending texts.

Since late elementary education is a critical period in the development of students’ reading strategies, the focus on older students is the main shortcoming of previous questionnaires. The RCSQ responded to this gap and tailored the items to this younger target group in a twofold way. First, just as the study of Merchie et al. (2014) on late elementary students’ text-learning strategies, the RCSQ items were formulated in a task-specific way. More specifically, the mental barrier was lowered by having students reflect on their strategy use in an authentic rather than hypothetical situation. Second, the wording of the items was adjusted to the target group by simplified language and in-depth consultation of experts, a primary teacher, and her students.

Further, this study explored the validity of a questionnaire measuring students’ reading strategy use in several ways. First, the well-grounded conceptualization and in-depth consultation of field experts contributed to the evidence for content validity. Second, construct validity was investigated by means of confirmatory factor analyses. Additionally, the measurement invariance tests also support the validity by revealing that diverse groups (i.e., according to students’ gender, general achievement level, and home language) did not respond significantly different on the RCSQ. If measurement invariance tests were conducted in the studies of the eight previous published measurement instruments on reading strategy use, the same results came to the fore (e.g., Mokthari and Reichard, 2002; Zhang, 2018a). Suggestions to further increase RCSQ’s validity are discussed later.

The second research objective was to examine individual differences in late elementary graders’ strategy use. As to these results, it is remarkable that students generally report to use metacognitive evaluation strategies more frequently, while the use of overt cognitive reading strategies are reported least often. This finding may possibly be explained by the students’ tendency to overestimate their actual strategy use in self-reports and a possible higher difficulty level of estimating one’s own metacognitive strategy use (Schellings and van Hout-Wolters, 2011). Notwithstanding, the value of self-report data should not be underestimated. They can provide insight into students’ perceptions of their strategy use and can serve as a reflection tool to make students aware of their strategy use or as starting point for interventions (Vandevelde et al., 2013).

The results concerning students’ individual differences in strategy use can also be related to previous research. First, gender differences in students’ strategy use have been examined several times, but findings are inconsistent, from reporting higher levels of metacognitive strategy use for boys (e.g., Zhang, 2018a), to reporting higher levels of diverse reading comprehension strategies for girls (e.g., Denton et al., 2015) or revealing no gender differences at all (e.g., Lindholm and Tengberg, 2019). Contrary to the latter, in our study, girls reported using more monitoring strategies than boys. As mentioned above, boys and girls do respond to the RCSQ in the same way (*cf*. measurement invariance tests), despite their reading strategy use differs.

Second, similar results were found for the student characteristics grade, achievement level, and home language. Both fifth-graders, low and average achievers, and non-native students reported using fewer evaluating strategies and more overt cognitive reading strategies than, respectively, sixth-graders, high achievers, and native students. Since the largest effect sizes were found when comparing high and low achievers’ strategy use, we will go into this in more detail. In line with our results, the literature review of Lin (2019) showed that high proficiency comprehenders use more metacognitive strategies (e.g., evaluating) than low proficiency comprehenders. Additionally, the study of Denton et al. (2015) indicated a higher score on evaluating strategies for older students and high achievers. Concerning cognitive strategies, our results contradict previous studies reporting either no significant difference in using cognitive strategies or a significant difference, with high achievers reporting a higher use of cognitive strategies than low achievers (Lin, 2019). However, our results can be consistent with the statement of Seipel et al. (2017) that poor and good readers differ in what strategies are used instead of making more/less use of all types of strategies. Further, Cromley and Azevedo (2006) suggested that low comprehenders systematically overestimate and high comprehenders systematically underestimate the frequency of their strategy use on a self-report, another possible explanation for our results.

### 6.1. Limitations and future directions

Limitations are inherently related to the current study and are considered below. First, self-report measures were used in order to collect data within a large group of students. However, measuring strategies through a self-reporting strategy is often criticized (e.g., Schellings and van Hout-Wolters, 2011). Therefore, future research should combine this data with other data collections methods (e.g., trace data; Bråten and Samuelstuen, 2007). A comparison of the results collected with different methods can additionally examine the construct validity of the RCSQ and examine possible over - or underestimation of self-reported strategies. Second, also the quality of the strategy use can be investigated in-depth (e.g., Denton et al., 2015). Third, the strategy use was measured at only one moment in time, and it would be interesting to include more measurement occasions for two reasons: (1) to obtain a more precise result independent of time-specific features or contexts and, (2) to map students’ strategy use evolution. Fourth, a larger pilot study including cognitive interviews could be recommended to assess the cognitive validity of the RCSQ items more in-depth (e.g., Vandevelde et al., 2013). Fifth, it would be interesting to verify whether the task-specific approach of the RCSQ, referring to a specific reading comprehension task, indeed generates more accurate results than a general instrument. Finally, future research is needed to explicitly examine the criterion validity of the RSCQ, for instance by studying the extent to which the RSCQ is related to reading comprehension outcomes. In this respect, it would also be valuable to evaluate the relationship between the RCSQ scores and other self-report measures (e.g., reading frequency, reading engagement). At present however, no standardized reading comprehension test is available in Flanders, especially not one focusing on comprehending expository texts.

### 6.2. Implications for research and practice

This study contributes in several ways to the current reading literature and practice. First, the RCSQ responds to the lack of a reading comprehension strategy measurement instrument attuned to the critical age of late elementary students. Furthermore, the society is – not only in Flanders but also worldwide – evolving toward an increasingly global and diverse society (e.g., Tenenbaum et al., 2017). By focusing on the strategy use of native, non-native, and bilingual students, the RCSQ can take into account this growing diversity. The RCSQ can be used by researchers to gain insights in the particular strategy use of native and bilingual students (e.g., the use of a home language as reading comprehension strategy). Teachers can also use the RCSQ to adapt their instruction on the specific strategy use of diverse student groups. Moreover, reading comprehension, including the use of appropriate reading strategies, is – despite its internationally recognized importance – a challenge for many students worldwide as well. Therefore, it seems valuable to test and use the RCSQ also in other countries. Further, the RCSQ is a valuable instrument for elementary teachers to adapt their instruction based on the mapped specific strategy use of diverse student groups. In addition, the developed questionnaire also offers great potential for upper elementary students to monitor their own reading comprehension strategy use.

In conclusion, the RCSQ can inform researchers, elementary school teachers, and students on reading comprehension strategy use, to better align instruction to students’ specific needs.

## Data availability statement

The raw data supporting the conclusions of this article will be made available by the authors, without undue reservation.

## Ethics statement

Ethical review and approval was not required for the study on human participants in accordance with the local legislation and institutional requirements. Written informed consent to participate in this study was provided by the participants’ legal guardian/next of kin.

## Author contributions

RB, EM, and HK designed the study. RB was in charge of the data collection and wrote the main part of the manuscript. YR provided in-depth assistance throughout the data analysis. All authors contributed throughout the different writing stages of the manuscript and approved the submitted version.

## Funding

This research was supported by a grant from the Special Research Fund of Ghent University.

## Conflict of interest

The authors declare that the research was conducted in the absence of any commercial or financial relationships that could be construed as a potential conflict of interest.

## Publisher’s note

All claims expressed in this article are solely those of the authors and do not necessarily represent those of their affiliated organizations, or those of the publisher, the editors and the reviewers. Any product that may be evaluated in this article, or claim that may be made by its manufacturer, is not guaranteed or endorsed by the publisher.
