# Supplementary material for: Development of the Reading Comprehension Strategies Questionnaire (RCSQ) for late elementary school students
Source: Front Psychol. 2023 Jan 4;13:1016761. doi: 10.3389/fpsyg.2022.1016761 (PMC9848395; doi:10.3389/fpsyg.2022.1016761)
Supplement: Supplementary file 1 [file Table_1.DOCX]

**Appendix**

Reading Comprehension Strategies Questionnaire (RCSQ)

| **Code** | | **Item** |
| --- | --- | --- |
| **Overt cognitive reading strategies** | | |
| OCOG1 | I tended to look up unknown words in the dictionary. | |
| OCOG2 | I marked or underlined the most important things to better understand the text. | |
| OCOG3 | I used scratch paper. | |
| OCOG4 | I wrote a summary to understand the text in a better way. | |
| OCOG5 | I made a schema. | |
| OCOG6 | I marked or underlined unknown words. | |
| OCOG7 | During reading, I made short notes to better understand the text. | |
|  |  | |
| **Covert cognitive reading strategies** | | |
| CCOG1 | Before reading, I first looked at the questions. | |
| CCOG2 | I read the first line of each paragraph to get the gist of the text. | |
| CCOG3 | I tried to understand the main idea in the text. | |
| CCOG4 | I made predictions as to what would follow next in the text. | |
| CCOG5 | While reading, I asked myself questions about the text to better understand the text. | |
| CCOG6 | When there were pictures, I looked at them to understand the text. | |
| CCOG7 | I tried to use what I already knew about the text topic to better understand the text. | |
|  | | |
| **Monitoring** | | |
| MON1 | ﻿While reading, I asked myself: “Am I doing well?” | |
| MON2 | ﻿While reading, I asked myself: “Do I still have enough time?” | |
| MON3 | ﻿While reading, I asked myself: “Is it working well this way?” | |
|  | | |
| **Evaluating** | | |
| EVA1 | I immediately knew how I could start solving the questions. | |
| EVA2 | ﻿While reading, I managed to stay attentive and concentrated. | |
| EVA3 | ﻿While reading, I made sure I understood everything. | |
| EVA4 | I did well in understanding the text. | |
| EVA5 | I have checked my test answers well. | |
| EVA6 | I think I have taken a good approach to understand the text. | |
|  |  | |
| **Using home language in view of comprehending texts*** | | |
| HL1 | I thought of words from my first language to find out the meaning of unknown words. | |
| HL2 | I marked or underlined unknown words and translated them into my first language. | |
| HL3 | I translated unknown words into my first language to understand the text better. | |

* Items HL1, HL2, HL3 were exclusively filled in by non-native and bilingual students. First language/home

language was considered as any language except Dutch.
